# Polysaccharide of Radix Pseudostellariae Improves Chronic Fatigue Syndrome Induced by Poly I:C in Mice

**DOI:** 10.1093/ecam/nep208

**Published:** 2011-06-23

**Authors:** Rong Sheng, Xianxiang Xu, Qin Tang, Difei Bian, Ying Li, Cheng Qian, Xin He, Xinghua Gao, Rong Pan, Chong Wang, Yubin Luo, Yufeng Xia, Yue Dai

**Affiliations:** ^1^Department of Pharmacology of Chinese Materia Medica, China Pharmaceutical University, Nanjing 210009, China; ^2^Department of Chinese Materia Medica Analysis, China Pharmaceutical University, Nanjing 210009, China

## Abstract

Radix Pseudostellariae is used as a tonic drug in traditional Chinese medicine with immunomodulating and anti-fatigue activities, and the polysaccharide is considered as the main active component. The purpose of this study is to examine the effect of the polysaccharide isolated from Radix Pseudostellariae (PRP) on mouse chronic fatigue syndrome (CFS) induced by intraperitoneal injection of polyriboinosinic:polyribocytidylic acid (poly I:C), a double-stranded synthetic RNA. It has shown that the fatigue symptom of mice lasted at least 1 week as evaluated by forced swimming time. PRP (100, 200, 400 mg kg^−1^), orally administered 3 days before poly I:C injection, showed dose-dependent anti-fatigue effects. In addition, poly I:C led to evident alternations in neuroendocrine and immune systems of mice, such as reduced spontaneous activity and learning ability, declined serum level of corticosterone, increased weight indexes and T lymphocyte numbers in thymuses and spleens, and increased CD4^+^/CD8^+^ ratio but decreased proliferation ability of T lymphocytes in spleens. PRP alleviated the abnormalities caused by poly I:C, and restored the function of hosts to normal conditions. The findings suggest that PRP is beneficial to CFS, and the underlying mechanisms of action involve neuroendocrine and immune systems.

## 1. Introduction

Chronic fatigue syndrome (CFS) was first defined in 1988 by the US Centers for Disease Control and Prevention as unexplained disabling fatigue lasting more than 6 months, together with a combination of non-specific accompanying symptoms [1]. CFS patients usually experience substantial reductions in occupational, educational and social functions, which collectively hamper the quality of life [2]. In addition, CFS is a debilitating syndrome with a cluster of multi-system dysfunctions [3], mainly involving the neuroendocrine and immune systems, and usually influencing other systems as well [4–7]. The pathogenesis of CFS probably includes immune system abnormalities, chronic immune activation, hypothalamic-pituitary-adrenal (HPA) axis dysfunction, brain anomalies, emotional stress and exogenous insults [8]. However, little is known about the underlying mechanisms of the fatigue symptoms in any clinical condition. Viral infection is of vital importance to CFS as considered from an etiologic aspect. Emotional stress appears to be important as well, as it impairs the normal function of the immune system against infections. Furthermore, emotional stress has been shown to determine, whether or not, an individual develops fatigue symptom upon virus infection, and can lead to the activation of the HPA axis. Likewise, a number of studies have focused on altered immune function in CFS, and immune function anomalies have been considered as potential explanations for the symptom complex or as possible predisposing factors for altered responses to infections.

Polyriboinosinic:polyribocytidylic acid (poly I:C), a double-stranded synthetic RNA, is frequently applied in animal experiments to mimic a condition of viral infection. Recently, Katafuchi et al. [9] reported that intraperitoneal injection of poly I:C in rats resulted in a significant and persistent reduction of spontaneous running wheel activity, which could be considered as an animal model of immunologically chronic fatigue with mechanisms closely related to the neuronal-endocrine-immune interactions.

Alternative medicines including Traditional Chinese Medicine have advantages of being effective to alleviate symptoms of CFS such as fatigue, disordered sleep, cognitive handicap and other complex complaints with minor side effects. The underlying mechanisms mainly include regulating immune function, adjusting abnormalities in the HPA axis and antioxidation [10]. *Pinus pinaste* bark could alleviate fatigue and activate the endogenous antioxidant enzyme system [11]. *Angelica sinensis* and *Matricaria chamomilla* extracts could ameliorate fatigue and sleep disturbances [12]. *Panax ginseng* has been used for centuries in Oriental folk medicine against fatigue. It could elevate movement ability and resist against CNS fatigue in mice [13]. Clinically, Ginseng could alleviate chronic fatigue, reducing fatigue severity and curtailing fatigue duration [14]. *Acanthopanacis senticosus* or *Siberian ginseng* showed ameliorating effects on CFS in a placebo-controlled clinical study [15]. *Crataegus oxyacantha* showed a significant increase in exercise time and an improvement in life quality in a three month placebo-controlled, randomized, double-blind trial [16]. *Eucommia ulmoides* leaves enhanced the 3-hydroxy-acyl-CoA dehydrogenase specific activity and lactate dehydrogenase specific activity in the skeletal muscle, suggesting that exercising and intaking of the leaf cooperatively could decrease the possibility of lactate accumulation in skeletal muscle and that the administration of *E. ulmoides* leaves along with low intensity training can enhance the ability of a muscle to resist fatigue [17] *Rhodiola rosea* is used as a central stimulant, antidepressant and antifatigue drug. *Rhodiola rosea* extract showed a significant improvement in the total mental performance [18] *Oryza sativa*, *Gentiana lutea symphyandra* and *Trichopus zeylanicus*-treated mice performed a better physical endurance in forced swimming tests [19–21]. *Withania somnifera*, Quercetin and *Hypericum perforatum L.* have been used for the treatment of CFS with the purpose of reducing lipid peroxidation, restoring the glutathione levels and increasing the superoxide dismutase levels in the brains of CFS mice [22]. *Ginkgo biloba* and *Vaccinium myrtillus* (bilberry) have also been reported containing beneficial antioxidants for CFS [23].

Radix Pseudostellariae, the roots of *Pseudostellaria heterophylla*, is widely used as a tonic drug in China for the treatment of splenic asthenia syndrome, which partially manifests features of CFS. Macromolecule polysaccharides have long been recognized as the main active component of Radix Pseudostellariae [24]. They possess antiinfectious, antioxidative and immunomodulating activities [25–27], suggesting that they might be beneficial to CFS. In the present study, the antifatigue effects of the polysaccharides were evaluated in poly I:C-induced mouse model of CFS. The mechanisms of action were addressed in terms of neurological, endocrine and immune regulations.

## 2. Materials and Methods

### 2.1. Chemicals and Reagents

Poly I:C was purchased from Guangdong BangMin Pharmaceutical Co., Ltd., Guangdong, China. Corticosterone radioimmunity kit was purchased from Beijing PuerWeiye Biotechnology, Ltd., Beijing, China. Concanavalin A (Con A) was purchased from Sigma, USA. Phycoerythrin (PE) antimouse CD4 (L3T4, Cat#: BM0404), PE antimouse CD8 (Lyt-2, Cat#: EM0804), fluorescein isothiocyanate (FITC) antimouse CD3 (Cat#: EM0301) and lysing solution were purchased from MultiSciences Biotech Co., Ltd., Shanghai, China.

### 2.2. Animals

Male ICR mice (18–22 g) were purchased from the experimental animal centre of China Pharmaceutical University. The animals were housed with free access to standard laboratory chow and water at 23 ± 2°C with a dark and light cycle of 12 h each. Animal experiments were conducted in accordance with current ethical regulations for institutional animal care and use of China Pharmaceutical University. The study was approved by the Institutional Ethical Committee of China Pharmaceutical University.

### 2.3. Preparation of PRP

Radix Pseudostellariae was purchased from Anhui Province, China, and was authenticated by Professor Mian Zhang, China Pharmaceutical University. Powdered crude drugs (400 g) were extracted three times by refluxing with 80% ethanol (1 L) at 90°C for 2∼3 h each time. After filtration, the gruffs were extracted again for three times with water (1.5 L) at 90°C for 2-3 h each time. The extracted solution was condensed to 400 mL and deproteinated by applying the Sevag method. The solution was then added to absolute ethyl alcohol until the ethanol concentration was 80% and kept overnight, followed by filtration. The precipitate was dissolved with water (100 mL) and then added absolute ethyl alcohol until the ethanol concentration was 80%, filtrated and repeated once again. The precipitate was washed with 95% ethanol, absolute ethyl alcohol and acetone by turns, and then dried at 50°C, leaving an ivory white powder. The yield ratio of the polysaccharides in the dried roots was 2.13%, and the purity was 72.65% as measured by a phenol-sulfuric acid colorimetric method with glucose as a reference. The crude dried PRP was dissolved in distilled water just before use.

### 2.4. Therapy Regimen

Fifty mice were randomly divided into five groups: 1: normal; 2: control; 3: PRP (100 mg kg^−1^); 4: PRP (200 mg kg^−1^); 5: PRP (400 mg kg^−1^). PRP was orally administered with a volume of 0.2 mL/10 g body weight at 8:00 am for consecutive 17 days. Normal and control mice were given orally an equal volume of vehicle. On day 4, except the mice in normal group, all mice were intraperitoneally injected poly I:C (5 mg kg^−1^) to induce CFS.

### 2.5. Forced Swimming Test

On day 1, 4, 7, 10 and 13 after injection of poly I:C, forced swimming test was carried out in a plastic pool (48 × 36 × 29 cm). The water depth and temperature were 20 cm and 22 ± 1°C, respectively. Mice were loaded 5% of the body weight of lead threads at the bases of the tails. They were then forced to swim until fatigue, defined as failure to rise to the surface of the water to breathe within an 8-s period. The time until fatigue was recorded.

### 2.6. Spontaneous Activity

On day 3 before injection, day 1 and 7 after injection of poly I:C, open field test was performed to evaluate the locomotive and explorative behavior in a novel circumstance. The open field apparatus was a cylindrical box (diameter: 30 cm, height: 20 cm), which was divided into 19 equal grids on the floor. Mice were placed individually on the centre of the apparatus, and were allowed to move freely for 5 min. During the test, the total number of cross points was manually recorded.

### 2.7. Learning Ability

On day 14 after injection of poly I:C, step-down test was performed to examine the learning ability of mice. The apparatus consisted of a transparent acrylic chamber with an electrifiable grid floor. A rubber platform (diameter: 4.5 cm, height: 4.5 cm) was placed on the grid floor. Mice were allowed to move freely in the chamber for 3 min without current in the electrifiable grid floor. In the following 5 min, mice were put on the platform with current in the electrifiable grid floor. If they stepped down from the platform (“error trial"), they were punished by an electric foot shock (36 V, AC). The error number was recorded as the final grade for the learning ability test.

### 2.8. Assay of Serum Corticosterone Level

On day 14 after poly I:C injection, mice of all groups were sacrificed, and the peripheral blood samples (0.2 mL per mouse) were collected. The blood samples were centrifuged for 10 min at 4°C, 3000 r min^−1^. Sera were stored at −70°C until assay. Corticosterone level was measured by radioimmunoassay according to the manufacturer's instructions.

### 2.9. CD4^+^ T and CD8^+^ T lymphocytes in Peripheral Blood

On day 14 after poly I:C injection, anticoagulated blood (0.2 mL per mouse) was collected, and stained with 5 *μ*L of PE anti-mouse CD4, PE antimouse CD8 and FITC antimouse CD3, respectively. The blood was incubated in the dark at 4°C for 1 h. Then, the lysing solution (2 mL) was added to lyse erythrocytes. The mixtures were incubated in the dark at room temperature for 30 min, and centrifuged at 4°C for 1500 r min^−1^. Then, they were washed with 2 mL PBS, and dissolved with PBS (500 *μ*L). The cells were analyzed on a flow cytometer, using cell quest software.

### 2.10. Weighing and Histological Analysis of Immune Organs

On day 14 after poly I:C injection, spleens and thymuses of mice in each group were removed and weighed, and the weight indexes (mg g^−1^ b.w.) were calculated. Then they were fixed in formaldehyde, sectioned and embedded in paraffin, and sliced for hematoxylin and eosin staining. Several parameters, including peripheral cortex thickness of thymus, numbers of splenic corpuscles and periarterial lymphatic sheaths, splenic sinusoid and splenic cord size, were evaluated in the histological analysis.

### 2.11. Spleen Cell Proliferation Assay

On day 14 after poly I:C injection, spleens of six mice in each group were gently homogenized and repeatedly pipetted to attain a single splenocyte suspension. The cells were washed twice with ice-cold PBS and separated by nylon mesh (cell viability > 95%), and then seeded in 96-well culture plates at a final concentration of 5 × 10^6^ cells/well in a RPMI-1640 medium supplemented with 10% FBS. Splenocytes, treated with or without mitogen Con A (5 *μ*g mL^−1^), were incubated for 68 h at 37°C in a 5% CO_2_ incubator. Subsequently, 20 *μ*L of MTT (3-(4,5-dimethylthiazol-2-yl)-2,5-diphenyltetrazolium bromide) (0.5 mg mL^−1^) was added into each well, and the cells were incubated for another 4 h. After that, the supernatant was removed and the formazan crystals were dissolved using DMSO (dimethyl sulphoxide) (150 *μ*L). The absorbance at 570 nm was read with a Model 1500 Multiskan spectrum microplate Reader (Thermo, Waltham, MA, USA).

### 2.12. Statistics

All values were expressed as mean ± SEM. Data were statistically analyzed by ANOVA followed by post hoc Tukey test. Differences were considered significant at *P* < .05.

## 3. Results

### 3.1. PRP Increased the Body Weights of Mice

**Figure 1:**
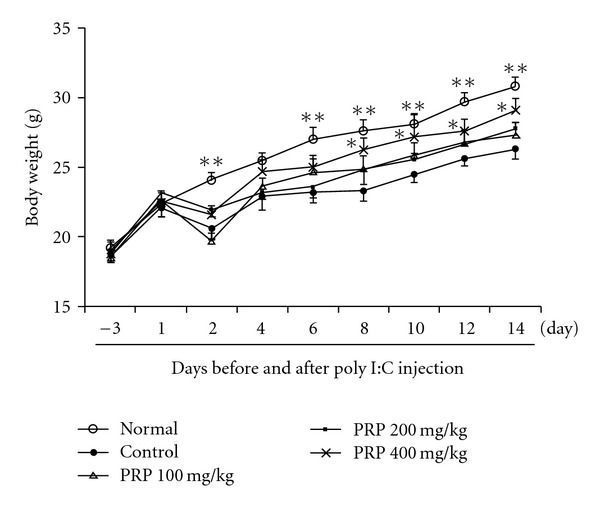
Effect of PRP on the body weights of mice treated with poly I:C. PRP was administered orally from 3 days before poly I:C injection for 17 days. Data are expressed as means ± SEM (*n* = 10). *P < .05; **P < .01 versus control.

Body weight, an indicator of health status, was measured every other day. Poly I:C injection resulted in a rapid and persistent decrease in the body weights of mice (Figure 1). PRP (100, 200, 400 mg kg^−1^) dose-dependently increased the body weights of mice during the 2 weeks after the injection of poly I:C. Statistically significant increase was observed in PRP (400 mg kg^−1^)-treated group from day 8 to 14.

### 3.2. Anti-Fatigue Activity of PRP

**Figure 2:**
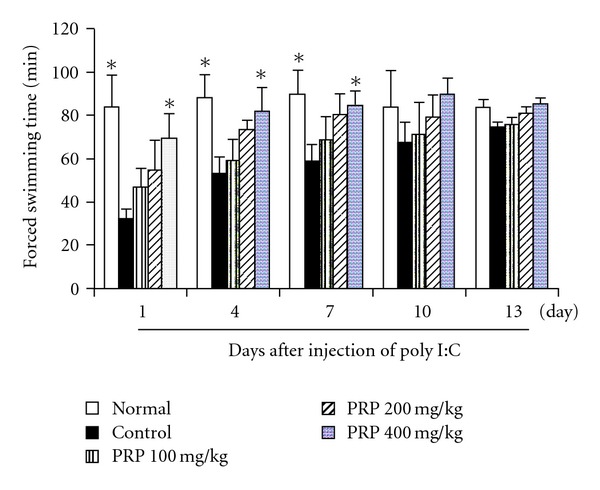
Effect of PRP on chronic fatigue induced by poly I:C in mice. PRP was administered orally from 3 days before poly I:C injection for 17 days. Forced swimming test was performed at indicated days. Data are expressed as means ± SEM (*n* = 10). *P < .05 versus control.

The forced swimming time of mice was considered as an indicator of fatigue. During 13 days of trial period, the forced swimming time of normal mice was within 80 to 90 min. At 6 h after poly I:C injection, the average forced swimming time of control group decreased sharply, and lasted at least for 1 week. From the second week, it gradually recovered, and yet, not reached the baseline level until day 13. PRP (100, 200, 400 mg kg^−1^) dose-dependently prolonged the forced swimming time during the full experiment period. Especially, PRP (400 mg kg^−1^) showed a statistically significant effect in the first week (Figure 2).

### 3.3. PRP Increased Spontaneous Activity

**Figure 3:**
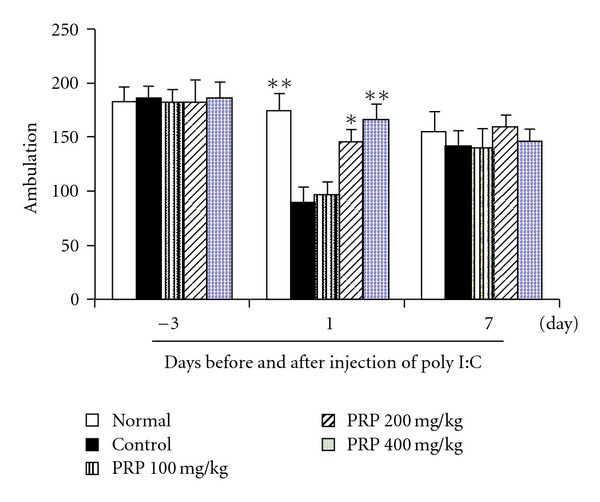
Effect of PRP on spontaneous activity in mice treated with poly I:C. Mice were placed individually on the center of the apparatus, and were allowed to move freely for 5 min. The total number of cross points was manually recorded at indicated days after injection of poly I:C. PRP was administered orally from 3 days before poly I:C injection for 17 days. Data are expressed as means ± SEM (*n* = 10). *P < .05, **P < .01 versus control.

Before and after poly I:C injection, the spontaneous activity of five groups of mice was examined by open field test. The results were presented in Figure 3. Baseline levels of spontaneous activities were of the similar level among all groups without significant differences. At 4 h after poly I:C injection, spontaneous activity of mice in control group decreased significantly compared with that of the normal group. PRP (100, 200, 400 mg kg^−1^) showed a dose-dependent increase in spontaneous activity. On day 7, the spontaneous activities of mice in both control and PRP-treated groups restored almost to the baseline level.

### 3.4. PRP Improved the Learning Ability

**Figure 4:**
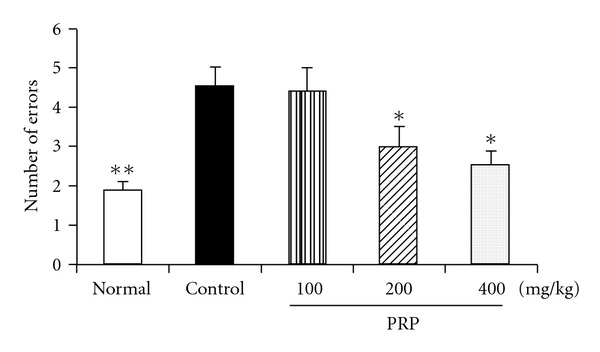
Effect of PRP on learning ability in mice treated with poly I:C. The error number of the learning training was recorded as the learning grade on Day 14 after injection of poly I:C. PRP was administered orally from 3 days before poly I:C injection for 17 days. Data are expressed as means ± SEM (*n* = 10). *P < .05 versus control.

On day 14 after poly I:C injection, the step-down latency of passive avoidance response (number of errors) of mice in control group was apparently longer than that of normal group, suggesting that poly I:C resulted in decline of learning ability. PRP (100, 200, 400 mg kg^−1^) treatment decreased the number of errors of mice in a dose-dependent manner. Statistically significant improvements of learning ability were displayed in PRP (200, 400 mg kg^−1^)-treated groups (Figure 4).

### 3.5. PRP Increased the Serum Corticosterone Level

**Figure 5:**
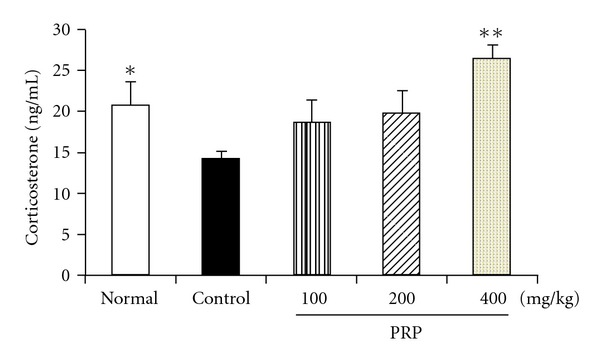
Effect of PRP on serum corticosterone level in mice treated with poly I:C. Peripheral blood samples were collected with 0.2 mL per mouse on Day 14 after injection of poly I:C. Serum corticosterone level was measured by radioimmunoassay assay. PRP was administered orally from 3 days before poly I:C injection for 17 days. Data are expressed as means ± SEM (*n* = 10). *P < .05, **P < .01 versus control.

As shown in Figure 5, the average serum corticosterone level of mice in control group was significantly decreased in comparison with normal group on day 14 after poly I:C treatment. PRP (100, 200, 400 mg kg^−1^) dose-dependently elevated the corticosterone level, and completely reversed the decrease of serum corticosterone at the dose of 400 mg kg^−1^.

### 3.6. Impacts of PRP on Immune Organ Index

**Figure 6:**
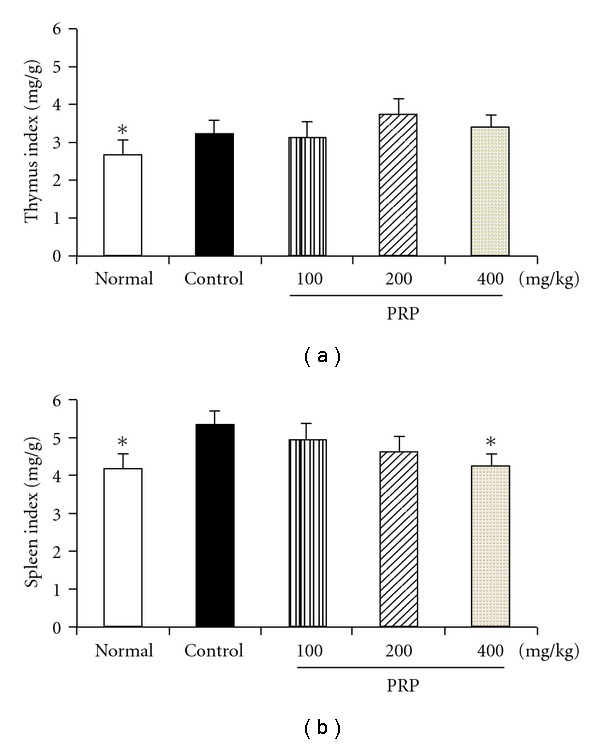
Effect of PRP on immune organ weight of mice treated with poly I:C. Mouse thymuses and spleens were removed and weighed on Day 14 after injection of poly I:C. Thymus and spleen indexes were calculated. PRP was administered orally from 3 days before poly I:C injection for 17 days. Data are expressed as means ± SEM (*n* = 10). *P < .05 versus control.

Poly I:C treatment resulted in the increases of both thymus and spleen indexes of mice. PRP (100, 200, 400 mg kg^−1^) did not affect the thymus index, but dose-dependently decreased the spleen index. The spleen index of mice in PRP (400 mg kg^−1^)-treated group was close to the normal value on day 14 after poly I:C injection (Figure 6).

### 3.7. Histological Findings of Immune Organs

**Figure 7:**
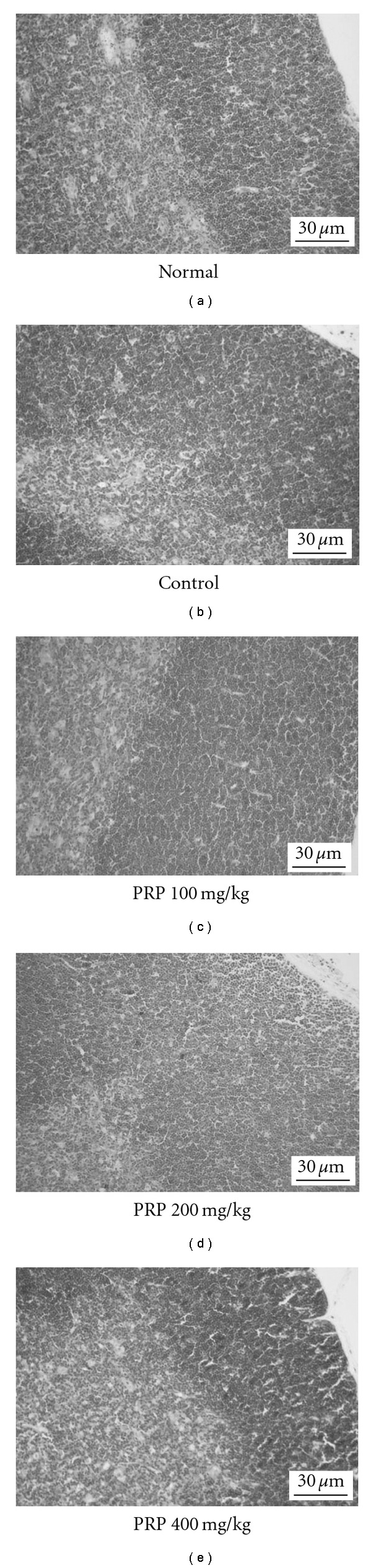
Representative histological changes in thymuses (hematoxylin and eosin, H&E, ×100). Thymuses were removed on Day 14 after poly I:C injection. Peripheral cortex thickness of thymus was evaluated.

Each thymus lobule has a peripheral cortex consisting of a dense population of thymocytes which surround the lightly stained central medulla. After treated with poly I:C, the thickness and the thymocyte numbers in the peripheral cortex of mouse thymus were obviously increased together with a compacted arrangement of thymocytes. PRP (100, 200, 400 mg kg^−1^) reduced the thymocyte population to normal level (Figure 7).

**Figure 8:**
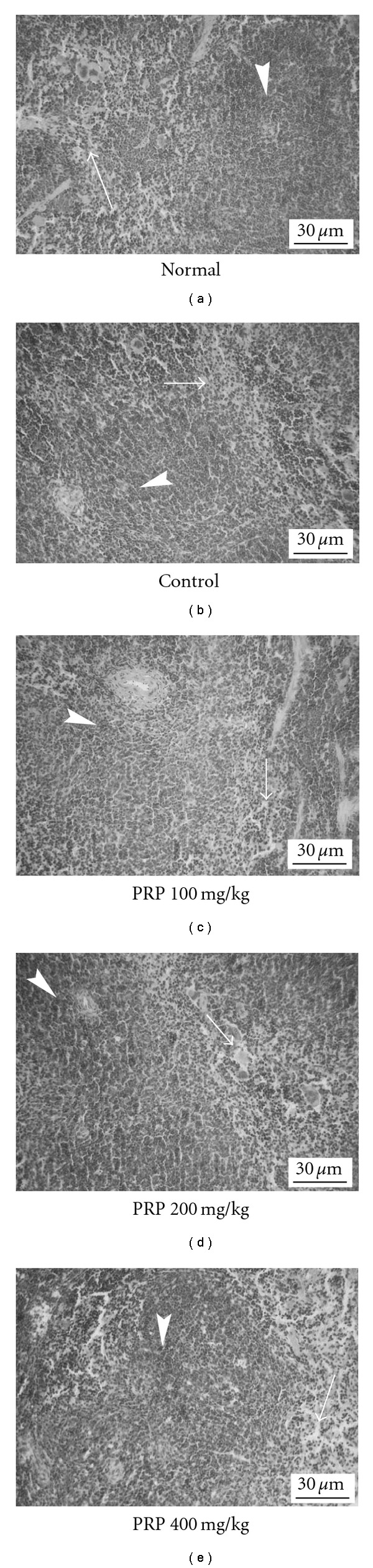
Representative histological changes in spleens (hematoxylin and eosin, H&E, ×100). Spleens were removed on Day 14 after poly I:C injection. Numbers of splenic corpuscles and periarterial lymphatic sheaths, splenic sinusoid and splenic cord size were evaluated.

There are white pulp, red pulp and marginal zone in spleen parenchyma. White pulp consists of splenic corpuscles and periarterial lymphatic sheaths. Red pulp consists of splenic sinusoid and splenic cord. Compared with normal group, size of both splenic corpuscles in white pulp and periarterial lymphatic sheaths in red pulp were obviously increased in control group, suggesting that poly I:C led to increase the T lymphocyte population in spleens. In addition, splenic sinusoid in red pulp distended and engorged slightly in control group. PRP (100, 200, 400 mg kg^−1^) ameliorated the histological changes in spleens to different degrees (Figure 8).

### 3.8. PRP Regulated the Ratio of CD4^+^ and CD8^+^ T Lymphocytes in Peripheral Blood

Peripheral blood of mice was collected on day 14 after poly I:C treatment for the detection of T lymphocyte population using flow cytometry analysis. It was shown that poly I:C resulted in a remarkable increase of CD4^+^/CD8^+^ ratio by increasing CD4^+^ T lymphocyte percentage and decreasing CD8^+^ T lymphocyte percentage. PRP (100, 200, 400 mg kg^−1^) modulated the percentages of the two types of lymphocytes and restored the CD4^+^/CD8^+^ ratio to normal (Table 1).

### 3.9. PRP Increased the Proliferation Ability of Spleen T Lymphocytes

**Figure 9:**
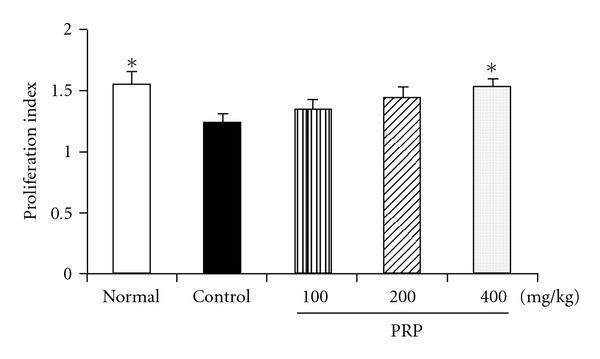
Effect of PRP on T lymphocyte proliferation of mice induced by Con A. PRP was administered orally from 3 days before poly I:C injection for 17 days. Splenocytes of mice were treated with or without mitogen Con A on Day 14 after injection of poly I:C. The proliferation ability was evaluated by MTT assay. Data are expressed as means ± SEM (*n* = 6). *P < .05 versus control.

The proliferation of mouse spleen T lymphocytes was induced by Con A *in vitro*. As shown in Figure 9, the proliferation ability of T lymphocytes in poly I:C-treated group was significantly reduced. PRP at doses of 100, 200 and 400 mg kg^−1^ promoted the proliferation ability, and the proliferation index in PRP (400 mg kg^−1^)-treated group was close to that of normal group.

## 4. Discussion

CFS is characterized by clinically unexplained fatigue, lasted at least for six months, which is not the outcome of ongoing exertion and is not substantially alleviated by rest. Although the exact pathogenesis of CFS remains unclear, current hypotheses postulate that special virus infection and immune dysfunction relevant to infection are contributed to the occurrence of CFS. For experimental purpose, several animal models of CFS have been established by injecting the bacterial antigen from *Corynebacterium parvum* and *Brucella abortus* [28–30], injection of poly I:C, and 15-day exposure to forced swimming [31]. Among these models mentioned above, poly I:C- or *B. abortus* antigen-induced fatigue is able to persist for more than 1 week in contrast to the short duration induced by *C. parvum* antigen. In addition, as CFS is not the result of ongoing exertion, fatigue due to forced swimming for 15 days seems to be an improper model of CFS. Therefore, in the present study, we evaluated the anti-CFS effect of PRP using poly I:C model, and explored the underlying mechanisms of PRP in terms of neuroendocrine and immune modulation.

Forced swimming time and spontaneous running wheel activity are usually adopted to represent the physical work capacity and fatigue condition of animals. In the current study, the forced swimming time of normal mice was nearly stable during the 2 weeks. Intraperitoneal injection of poly I:C resulted in a persistent decline of mouse swimming time, indicating that it can induce chronic fatigue condition in mice as in rats [9]. PRP, orally administered prior to poly I:C injection, prolonged mouse swimming time in a dose-dependent manner. Further, we found that poly I:C treatment led to a marked reduction of spontaneous activity of mice in the open field test by near 50% as compared with the normal group, and PRP exhibited ameliorating effect on the spontaneous activity in poly I:C-treated mice. The findings demonstrated that PRP had beneficial effects on chronic fatigue.

It is generally accepted that the persistent fatigue is not due to peripheral problems such as muscular weakness and muscle or joint pain, but involves complex central mechanisms [32]. Under the condition of chronic fatigue, cytokines produced in the brain exert various central actions, including activation of sympathetic nervous system and hypothalamic-pituitary axis, impairment of learning memory and influence of peripheral cellular immunity [33]. It has been reported that not only personal activity reduction, but cognitive disturbances are also exhibited in CFS patients [2]. Poly I:C is thought to induce central fatigue through modulating IFN-*α* and 5-HT expressions in the brain [34]. In the current study, it remarkably impaired the cognitive and learning ability of mice in the step down test. PRP could dose-dependently reverse the learning ability decline caused by poly I:C. Since PRP is a soluble micromolecule, it is probably difficult to cross the blood-brain barrier (BBB) and activate gliacytes directly. PRP may passively cross the BBB at “leaky" regions where the BBB is not intact, such as the circumventricular organs (CVO's), and directly act on endothelial cells of brain vasculature or glial cells in the CVO's, inducing the synthesis/release of central cytokines. On the other hand, PRP may activate immune system to release a lot of cytokines, which affect central nervous system indirectly.

The HPA axis carries out the functions to maintain homeostasis between physical and psychological stress. There are a few reports demonstrating that the basal serum cortisol level was below average in CFS patients [35]. In the present study, poly I:C treatment led to a remarkable decline of mouse serum corticosterone level. PRP elevated the serum corticosterone level in a dose-dependent manner, suggesting that it can modulate the function of HPA axis under the condition of chronic fatigue.

CFS patients have specific immune dysfunction profiles characterized by activation of lymphoid populations but suppression of some immune responses [36, 37]. The immune system of CFS patients reacts as if it is continuously combating an infection no matter whether an infectious agent is actually present at the time. But when the patients are challenged with stressors such as infections, immune responses may be blunted due to the production of anti-in*ﬂ*ammatory cytokines [38]. Mihaylova et al. [39] indicated that this pattern of dysfunction comprised a hyperin*ﬂ*ammatory resting state and targeted immunosuppression of responses. Substantial evidence has suggested that most of CFS patients have one or more immunological abnormalities related to infectious disorders [40]. In the present study, poly I:C led to a significant increase of both weights and lymphocyte numbers in thymuses and spleens, two important immune organs, suggesting that it might induce a persistent status of immune activation in mice. Moreover, T lymphocyte, an important effector cell in acquired immunity, can be divided into two subclasses, CD4^+^ T cells and CD8^+^ T cells. Of which, CD4^+^ T cells assist and induce the activation of T cells and B cells, while CD8^+^ T cells impede the activated B cells producing antibody. The CD4^+^/CD8^+^ ratio is an important indicator for assessing the function of cell-mediated immunity. Patients with autoimmune diseases have high CD4^+^/CD8^+^ ratio in their blood, indicating that imbalance between T lymphocyte subclasses could impair the immune system and result in diseases. Generally, poly I:C is recognized by toll-like receptor 3 located at the antigen presenting cells (APC). The ligand-receptor recognition leads to IFN-*α*/*β* production, followed by antigen-specific T cell activation and differentiation into effector cells. In the present study, poly I:C led to a remarkable increase of CD4^+^/CD8^+^ ratio in peripheral blood of mice by up-regulating CD4^+^ T cells and down-regulating CD8^+^ T cells, which further demonstrates that poly I:C could induce chronic immune activation, a typical pathological feature of CFS patients. In addition, T lymphocytes derived from spleens of poly I:C-treated mice showed hyporesponsiveness to the stimulation of Con A, indicating that poly I:C actually induced a suppression of immune responses. PRP significantly ameliorate the immunological anomalies caused by poly I:C through down-regulating spleen index, lymphocyte numbers and CD4^+^/CD8^+^ ratio in the peripheral blood as well as reversing the hyporesponsiveness of T cells in spleens.

**Figure 10:**
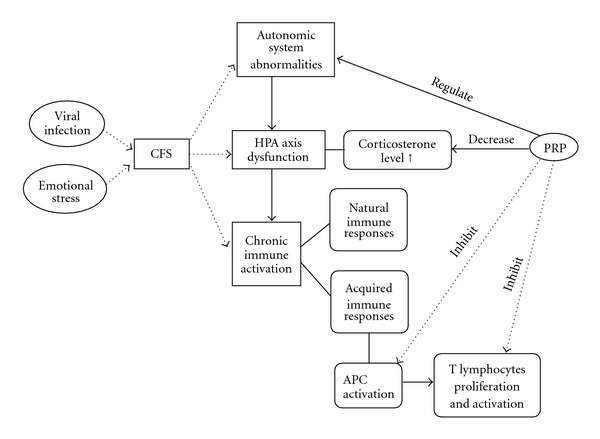
The hypothetical mechanisms of PRP against CFS in views of neurological, endocrine and immune modulation.

In conclusion, intraperitoneal injection of poly I:C would lead to a chronic fatigue status in mice, as mainly demonstrated by persistent decrease of forced swimming time and spontaneous activity, which was accompanied by neuroendocrine and immunological abnormalities. PRP effectively alleviated the chronic fatigue, and showed a potential preventive and therapeutic effect for human CFS (Figure 10).

## Figures and Tables

**Table 1:** Effect of PRP on CD4^+^ and CD8^+^ T lymphocyte populations in peripheral blood of mice treated with poly I:C.

Groups	Dose (mg kg^−1^)	CD4^+^/CD3^+^ (%)	CD8^+^/CD3^+^ (%)	CD4^+^/CD8^+^
Normal	—	66.8 ± 1.1*	29.8 ± 4.0	2.4 ± 0.3*
Control	—	74.2 ± 2.2	24.1 ± 1.6	3.5 ± 0.2
PRP	100	72.3 ± 0.7	24.7 ± 1.2	2.9 ±0.1
	200	71.3 ± 3.0	25.4 ± 2.0	2.9 ± 0.3
	400	71.5 ± 1.1	26.1 ± 0.7	2.7 ± 0.1*

Anticoagulated blood 0.2 mL of each mouse was collected on day 14 after injection of poly I:C. The cell populations were analyzed by flow cytometry. Data are expressed as means ± SEM (*n* = 5). *P < .05 versus control.
